# Trans-septal Mattress Suture: Securing Naso-Endotracheal Tubes With Enhanced Stability and Aesthetic Preservation

**DOI:** 10.7759/cureus.72670

**Published:** 2024-10-29

**Authors:** Anwesha Pattnayak, Rajat Mohanty

**Affiliations:** 1 Department of Oral and Maxillofacial Surgery, Kalinga Institute of Medical Sciences, Bhubaneswar, IND

**Keywords:** airway, anesthesia, intubation, maxillofacial surgery, nasal septum, naso-endotracheal, necrosis, suturing

## Abstract

Securing the endotracheal tube is vital for airway patency, especially in oral surgeries; naso-endotracheal intubation is ideal, but improper fixation risks nasal injury or accidental extubation. This technical note unveils the trans-septal mattress suture technique as a novel approach to secure naso-endotracheal tubes, amplifying stability, reducing trauma, and elevating aesthetic outcomes in head and neck surgeries. This study was performed at the Department of Oral and Maxillofacial Surgery at Kalinga Institute of Medical Sciences, Bhubhaneshwar, Khordha, Odisha, India, from December 2022 to March 2024 with due consent taken from all patients. Naso-endotracheal tube dislodgement during jaw fracture surgeries can cause nasal mucosa trauma and inadequate ventilation. Head and neck oncology cases require secure tube fixation due to prolonged intubation. Adhesive tapes lose adhesiveness, and fixators may obstruct visibility, while sutures offer reliable stabilization. A trans-columellar suture initially anchors the tube, but prolonged surgery risks skin necrosis and nasal deformity. Hence, we advocated a trans-septal mattress technique that relocates the knot to the septal area, preventing necrosis, ensuring stability, and minimizing postoperative deformity. Our trans-septal suture technique secures the naso-endotracheal tube by avoiding columellar scarring, ensuring better cosmesis while minimizing epistaxis risks. It offers a stable, cost-effective airway solution, ideal for prolonged surgeries, balancing function with aesthetics effectively.

## Introduction

The art of airway management stands as a cornerstone in the care of critically ill patients and trauma victims and the administration of anesthesia for surgeries and procedures both within and beyond the confines of the operating theater. In this regard, anesthesiologists skillfully select and deploy various techniques, including bag-mask ventilation, simple airway insertions into the oral or nasal cavities, advanced supraglottic airway devices, and more invasive approaches such as oral or nasotracheal intubation, percutaneous dilated cricothyroidotomy, or tracheostomy, all tailored to the patient’s clinical status and surgical needs. Nasotracheal intubation is particularly favored for surgeries of the head, neck, and oral cavity, or when securing the airway is paramount in trauma cases to prevent further injury. By threading the endotracheal tube through the nasal passage into the trachea, this technique offers enhanced stability and fixation compared to oral intubation due to the narrower, more controlled route provided by the nasal cavity. As a result, this method is highly esteemed not only by anesthesiologists but also by surgeons specializing in head and neck procedures, particularly in oropharyngeal, dental, and maxillofacial surgeries. Its ability to maintain a secure airway while offering an unobstructed surgical field and improving visibility makes it the preferred choice in such intricate operations. [1] Securing the endotracheal tube and ensuring airway patency is crucial during any general anesthesia procedure. When the surgical area is distant from the head and neck, stabilizing the endotracheal tube is relatively straightforward. However, in oral and maxillofacial surgeries - especially when intraoperative access to the dental occlusion is needed - fixing the endotracheal tube can present a challenge. In these situations, naso-endotracheal intubation is preferred, as it maintains airway patency while providing maximum head mobility for the patient. However, if the naso-endotracheal tube is not properly secured, it can result in nasal soft tissue injuries or accidental extubation [2,3]. The present technical note depicts the trans-septal mattress suture technique as a novel method for securing naso-endotracheal tubes in oral and maxillofacial surgeries, with the goal of reducing nasal trauma, preventing tube dislodgement, and minimizing postoperative deformities.

This technical note evaluates the technique's effectiveness in providing stable airway management, particularly in prolonged surgeries, while ensuring improved cosmetic outcomes and minimizing complications such as epistaxis and skin necrosis compared to traditional methods. The nasal cavity elegantly spans from the anterior nares, commonly known as the nostrils, to the posterior extremity of the nasal septum. Here, it transitions into the nasopharynx through the posterior nasal apertures, referred to as the choanae, which serve as a gateway connecting the nasal cavity to the throat. The primary blood supply nourishing this intricate structure is sourced from the sphenopalatine artery, a vital continuation of the maxillary artery. This artery forms a crucial anastomosis with the superior labial artery and the ascending branch of the greater palatine artery, converging within the anterior portion of the nasal septum to create a network known as Kieselbach’s plexus. This delicate, richly vascularized area of mucosa is termed Little’s area and is the most frequent site for spontaneous nosebleeds or epistaxis. However, it is essential to acknowledge that the entire nasal mucosa is abundantly supplied with blood, extending throughout the nasal cavity. As a result, even minor trauma to any region of this vascular tissue can lead to rapid and significant bleeding [4]. Our technique based on this anatomic knowledge keeps away from the plexus of vessels in the anterior nasal cavity, which prevents bleeding.

## Technical report

Various techniques have been implemented throughout history to secure a naso-endotracheal tube to avoid the complication of tube kinking or accidental extubation during surgical manipulation. One of the techniques for securing the tube is suturing it in the columella of the nose. Prolonged surgical duration and excessive pressure over the area of the sutures can lead to pressure necrosis of the columella, which in turn leaves an ugly scar over the nose.

Hence, to overcome this, we developed a suturing technique that can aid in sturdy grip over the tube by minimizing the complications. A mattress suture using a conventional cutting needle 2-0 silk thread through the trans-septal area allows the knot to be placed in the septal area, instead of the skin of the columella, thereby eliminating the complication of pressure necrosis of the skin.

This study was performed at the Department of Oral and Maxillofacial Surgery at Kalinga Institute of Medical Sciences, Bhubhaneshwar, Khordha, Odisha, India, from December 2022 to March 2024 with due consent taken from all patients.

Indications of the procedure include maxillofacial trauma involving occlusal discrepancy, orthognathic surgeries, and head and neck cancers not involving the nasal cavity.

Contraindications include nasoorbitoethmoid fractures, nasal polyps, and any tumors involving the nasal cavity and nasopharynx.

**Figure 1:**
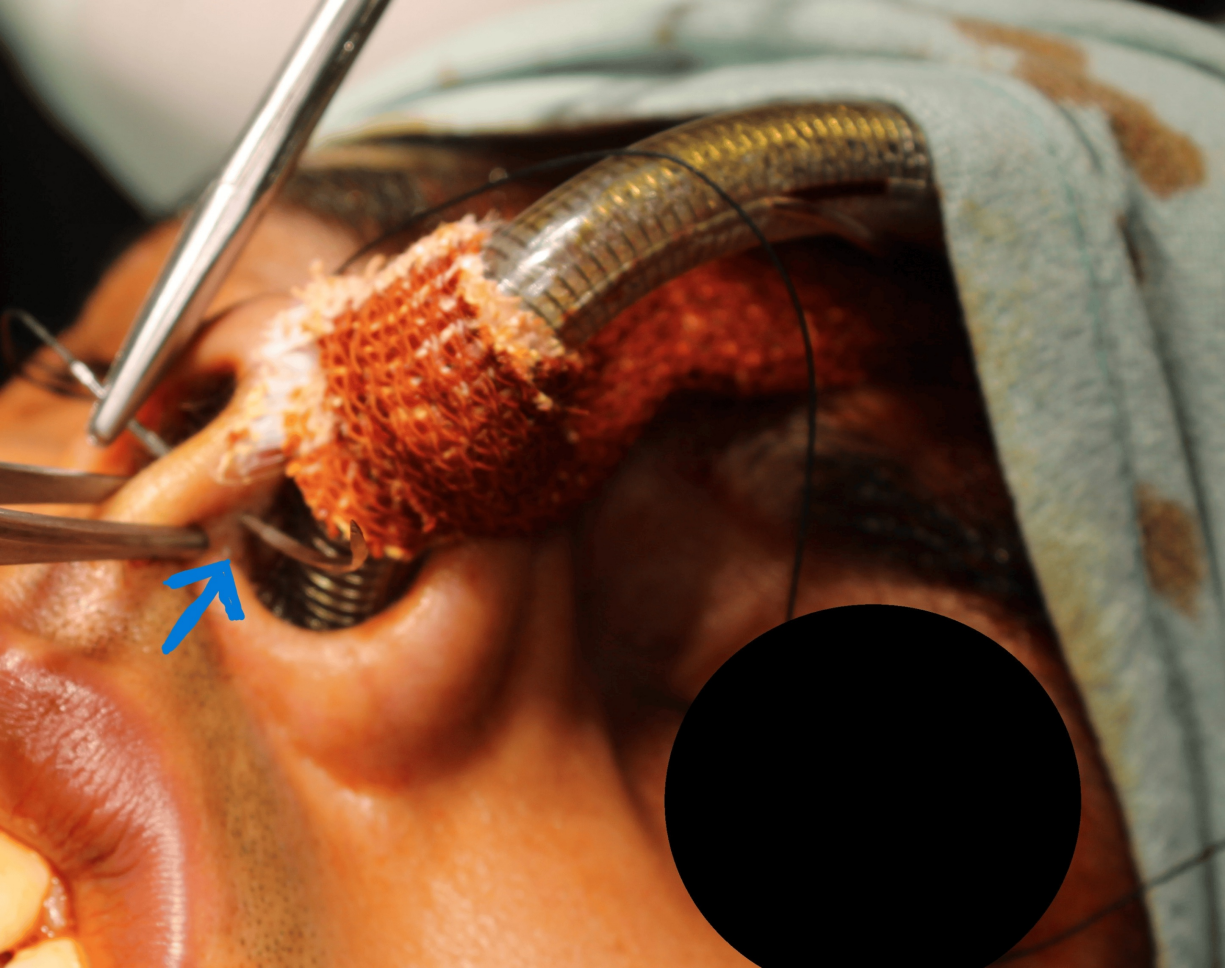
First Bite Through the Nasal Septum

An elastic adhesive bandage is used to initially secure the tube post intubation. The first bite of the suture is taken from the opposing nostril of the tube through the nasal septum. A non-toothed forceps is used to catch hold of the columella for retraction while taking the bite. Upon exiting the suture through the tube nostril, care should be taken not to damage the endotracheal tube (Figure 1).

**Figure 2:**
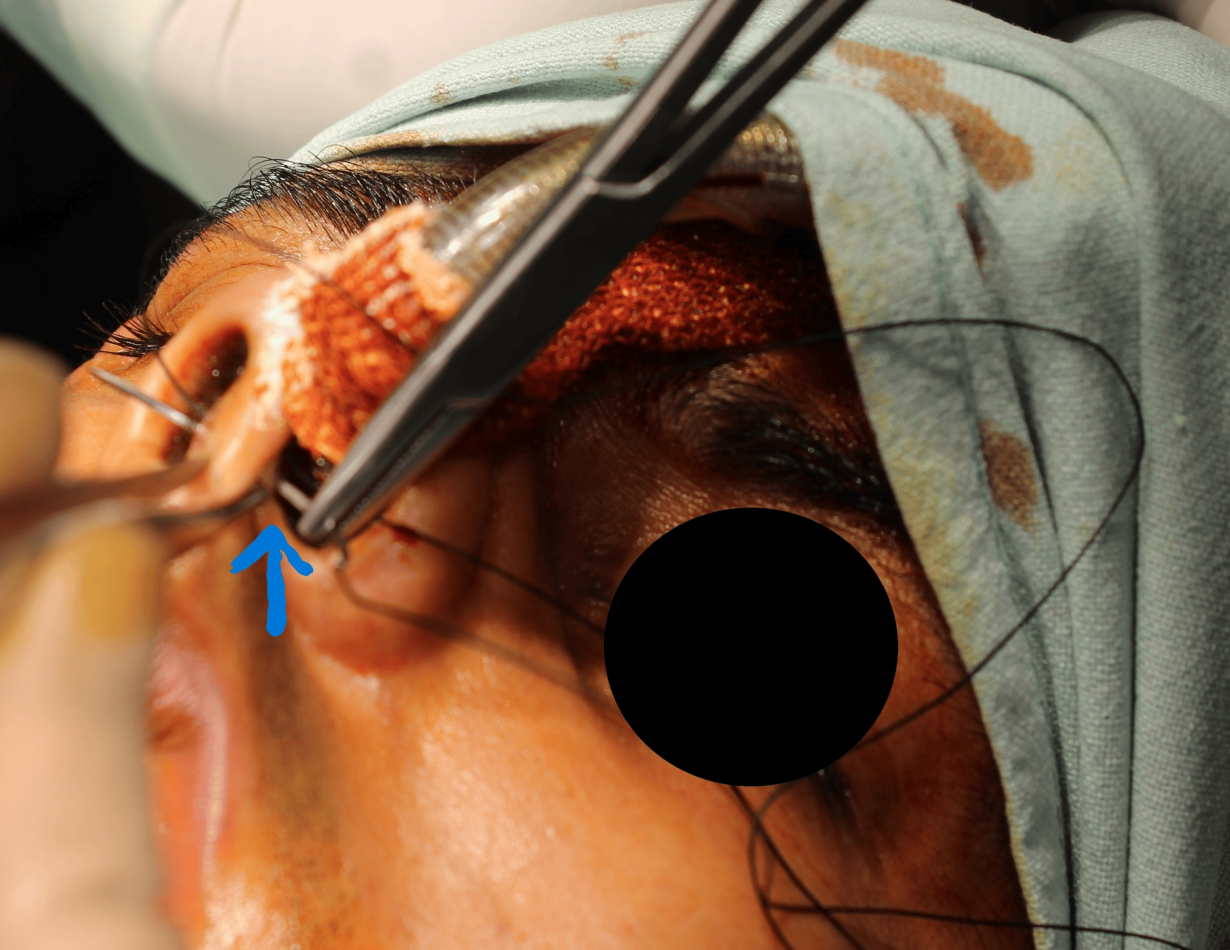
Second Bite Through the Nasal Septum

In a similar manner, the second bite is taken from the tube nostril to the non-tube nostril (Figure 2).

**Figure 3:**
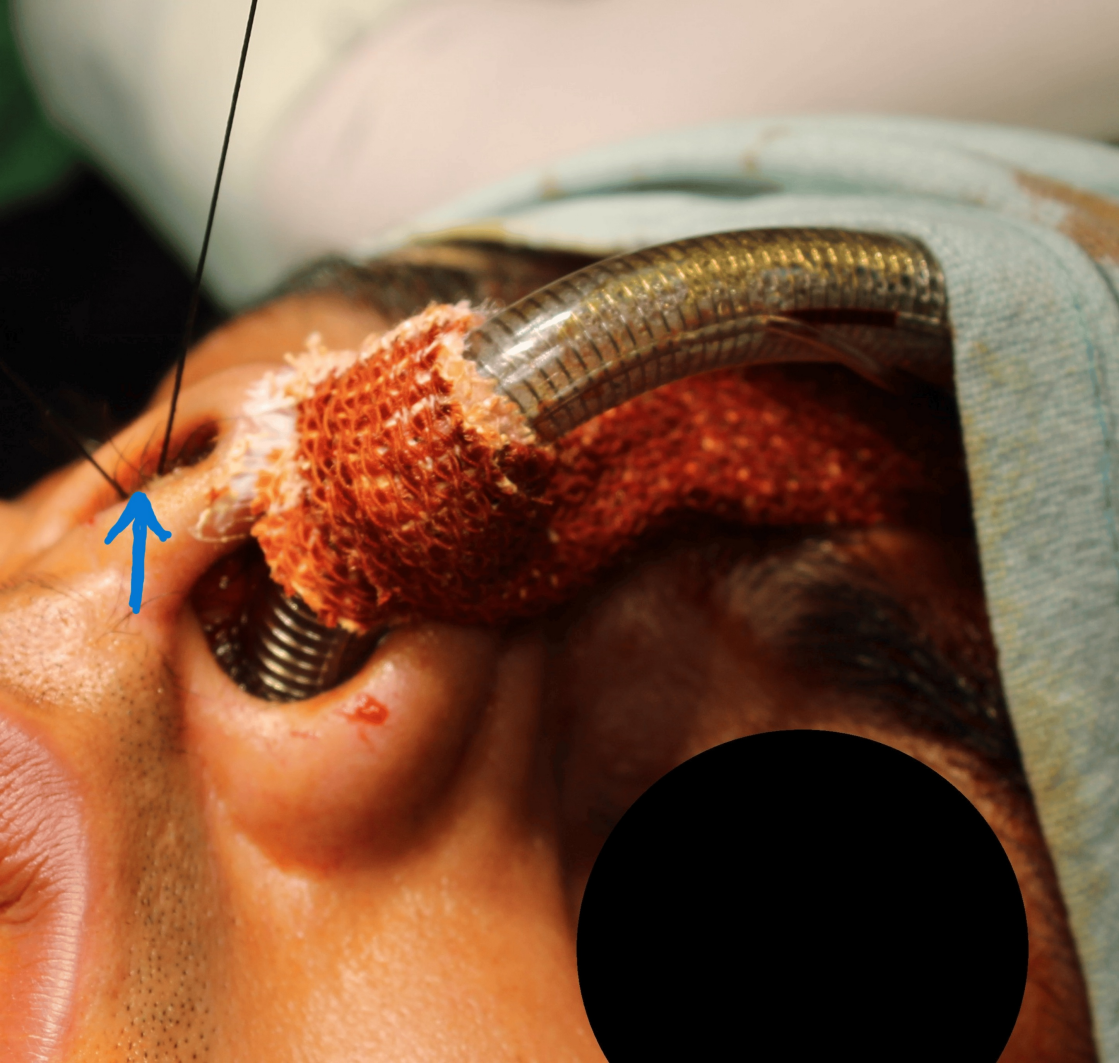
Securing the Knot Over the Mucosal Aspect

This mattress suture allows the knot to lie on the septal area, thereby preventing the risk of scarring (Figure 3).

**Figure 4:**
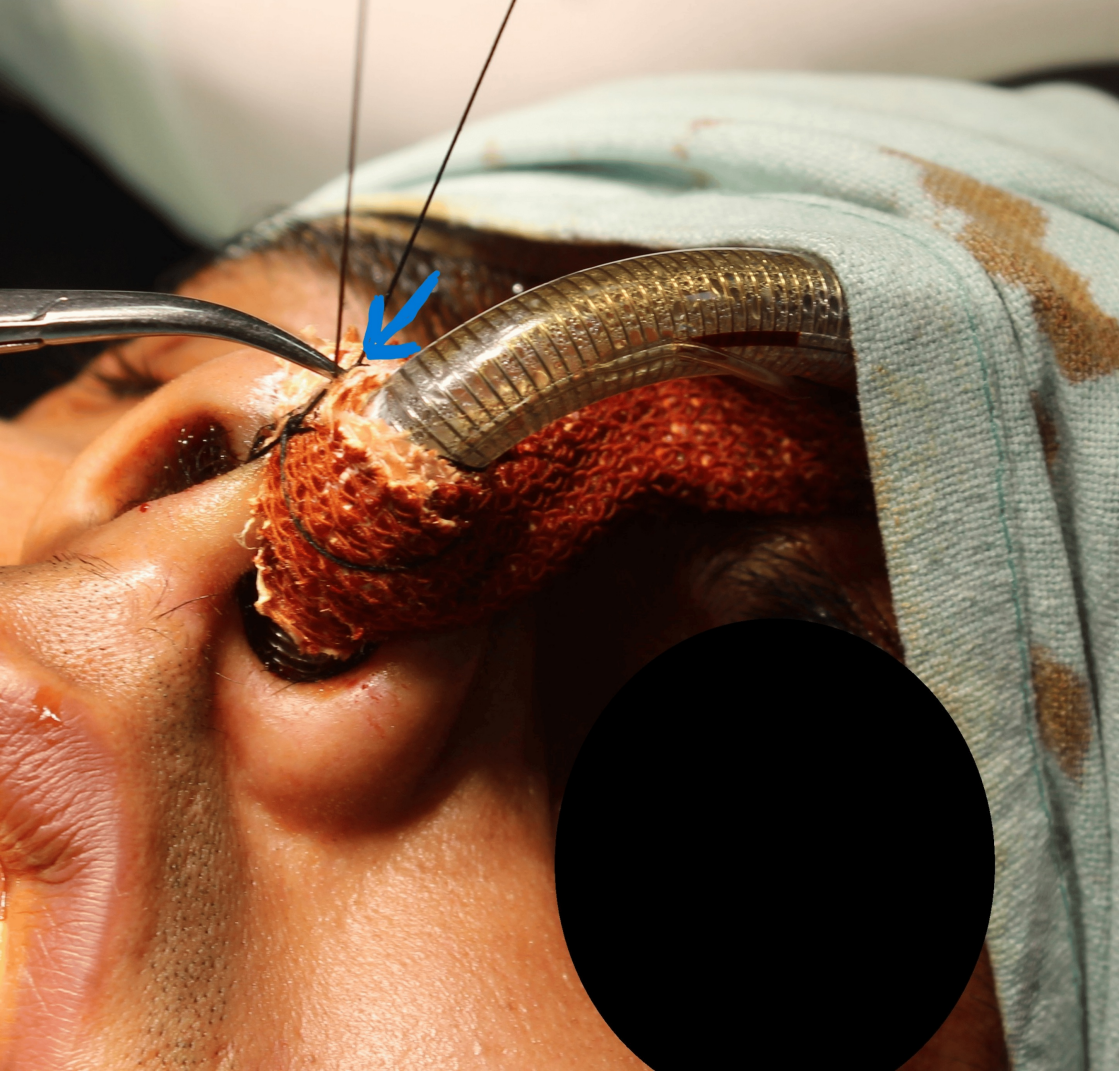
Securing the Naso-Endotracheal Tube

After the first knot had been secured over the adhesive tape, the longer end of the suture was circled around the tube once, and then the second knot was positioned over the tube sparing the skin of the nose. While doing so, the naso-endotracheal tube must be pushed anteriorly from behind to position the tube in its correct place. The third knot was positioned after an anti-clockwise rotation of the suture threads over the tube. The ends of the sutures were cut adequately, and the position of the tube was re-evaluated (Figure 4). The suture was removed post procedure, and the elastic adhesive bandages were kept in place till extubation.

Thus, this technique allows for surpassing the knots from the skin of the nose, thereby avoiding post-operative deformity.

## Discussion

Naso-endotracheal intubation is the most preferred technique in the field of oral and maxillofacial surgery. Unlike oral intubation, it provides the surgeon with a clear field of the interested surgical site in the head and neck area. Hence, access to the structures of the oral cavity is increased, aiding in precise surgery by direct visualization. Nasotracheal intubation, commonly used in orthognathic surgeries, enables easy maxillomandibular fixation with minimal complications. Securing the tube to the forehead with a well-applied head wrap is essential to prevent dislodgement and reduce the risk of pressure necrosis, especially during prolonged procedures involving hypotensive anesthesia, ensuring patient safety throughout surgery [5].

Surgery in the head and neck region necessitates various degrees of head manipulation and movement to ensure proper access, underscoring the importance of firmly securing the endotracheal tube [6]. Manipulations involved in jaw fractures for achieving accurate anatomical reduction and establishing functional occlusion cause either dislodgement of parts of the naso-endotracheal tube or partial expulsion of the tube. This causes trauma to the nasal mucosa by breaching it along with inadequate ventilation. In head and neck oncology cases, the longer surgical duration and the need for prolonged intubation demand fixation of the naso-endotracheal tube. Thus, preventing inadvertent movement that might result in patient extubation or damage to the nasal tissues is why it is universally acknowledged that securing the endotracheal tube securely is essential [6]. These tubes are secured using various methods, such as adhesive tapes around the tube, an external tube fixator device, cloth-based tapes, sutures, etc. [7]. Elias et al., in their article, elucidated a straightforward yet efficacious methodology for securing the nasotracheal tube with wire support, facilitating unimpeded head movement during surgery while reporting minimal complications across 197 patients over five years [2].

Sutures are one of the foolproof methods to secure the tube and manage to handle various degrees of movement during the intra-operative period. Most preferred suture materials include the use of braided silk sutures with larger diameters such as 2-0 silk sutures. For securing the tube, a trans-columellar suture is introduced, and the first knot is placed over the columellar skin. Subsequent knots are placed around the tube in a clockwise and anti-clockwise manner to achieve a firm hold over the tube. This first knot placed over the columellar skin tends to undergo pressure necrosis of the skin as it is subjected to a long duration of maxillofacial surgeries, resulting in post-operative scarring and deformity over the nose. Mahalle et al.'s study, involving 30 patients (80% male, 20% female), revealed significant differences in NETT displacement (p=0.031) and nasal tip trauma (p=0.049), favoring the flower stitch method for enhanced stability and reduced complications [6]. Adhesive tapes are most commonly used for fixing the tube in its place, but by themselves, they hold the disadvantage of losing their adhesiveness during scrubbing and painting of the head and neck areas. External tube fixators can be heavy and may obstruct the surgeon’s visual field. Fenje et al. elucidated a comparative assessment of adhesive bonds for various tapes securing endotracheal tubes, revealing significant disparities in adhesive strengths across tape types and tube materials [8].

Koshika et al., in their article, mentioned the use of a 3D printer to fabricate a proprietary device for fixing nasotracheal tubes in 335 patients undergoing oral and maxillofacial surgeries. No complications or tissue damage occurred, ensuring safety, surgical ease, and minimal invasiveness. Its firm immobilization prevents tube dislodgement and disconnection, regardless of anesthesiologist proficiency, enhancing overall intraoperative safety [9]. Iwai et al. highlighted the risk of nasal pressure sores from prolonged nasotracheal intubation, particularly in surgeries exceeding 10 hours, with a prevalence of 0.59%. Although medically minor, these sores cause cosmetic dissatisfaction. The use of hydrocolloid dressing (Duo Active® CGF) as a cushion between the tube and nasal skin has successfully prevented necrosis and ulceration in over 500 patients [10]. Thus, these comprehensive studies have meticulously evaluated both the advantages and limitations of their respective techniques for securing the naso-endotracheal tube.

Since, each methodology is accompanied by its distinct array of merits and drawbacks, one such disadvantage of securing the tube through a trans-columellar approach is the risk of pressure necrosis, resulting in scarring, which yields an unsatisfactory cosmetic outcome. Our approach via a trans-septal suture secures the knot over the septum, which avoids scarring of the columellar area, thereby improving cosmesis. Keeping in mind, the anatomy of the septum and the course of a septal branch of the superior labial artery, which is notorious for epistaxis, our suturing method does not bleed as anticipated. Therefore, our technique aims to provide a secure and stable airway while minimizing complications. However, if improper knotting techniques are used, the security of the knot and tube may be compromised.

## Conclusions

Following functionality, aesthetics is also of paramount importance; hence, every tiny detail pertaining to the patient should be taken into consideration. With a myriad of options available for the fixation of endotracheal tubes, the suturing option allows the surgeon and the anesthetist an effective way to perform longer-duration surgeries without compromise. Despite the advent of numerous devices, suturing remains a cost-effective and widely accessible method. Our technique of a trans-septal suture eliminates one of its cosmetic drawbacks and hence forms a cheap solution for securing the naso-endotracheal tube. Further research is encouraged in this technique to isolate potential flaws and to demonstrate further the effectiveness of our technique.
